# Spontaneous colitis in IL‐10‐deficient mice was ameliorated via inhibiting glutaminase1

**DOI:** 10.1111/jcmm.14471

**Published:** 2019-06-18

**Authors:** Jing Li, Lugen Zuo, Yun Tian, Yifan He, Zhichao Zhang, Pu Guo, Yuanyuan Ge, Jianguo Hu

**Affiliations:** ^1^ Department of Clinical Laboratory First Affiliated Hospital of Bengbu Medical College Bengbu Anhui China; ^2^ Anhui Key Laboratory of Tissue Transplantation Bengbu Medical College Bengbu China; ^3^ Department of Gastrointestinal Surgery First Affiliated Hospital of Bengbu Medical College Bengbu Anhui China; ^4^ Department of Oncology Shanghai Dermatology Hospital, Tongji University Shanghai China; ^5^ Tongji University Cancer Center The Shanghai Tenth People's Hospital, Tongji University Shanghai China; ^6^ Clinical Medicine of Bengbu Medical College Bengbu Anhui China; ^7^ Department of Colorectal Surgery The Third Affiliated Hospital of Nanjing University of Chinese Medicine Nanjing China

**Keywords:** BPTES, Crohn's disease, glutaminase1, intestinal barrier, T‐cell subsets

## Abstract

Immunity imbalance and barrier damage in the intestinal mucosa are the main pathogenic factors of Crohn's disease (CD). Bis‐2‐(5‐phenylacetamido‐1,2,4‐thiadiazol‐2‐yl) ethyl sulfide (BPTES) is a glutaminase 1 (Gls1) inhibitor with the dual functions of increasing glutamine levels and immune regulation. In this study, we focused on the role of BPTES in CD‐like enteritis and the possible mechanisms. We found that Gls1 expression was significantly increased in CD intestinal tissue compared with control tissue. Bis‐2‐(5‐phenylacetamido‐1,2,4‐thiadiazol‐2‐yl) ethyl sulfide treatment significantly ameliorated chronic colitis in the IL‐10^−/−^, as manifested by decreased disease activity index, body weight change, histological inflammatory degree and inflammatory cytokine expression. Bis‐2‐(5‐phenylacetamido‐1,2,4‐thiadiazol‐2‐yl) ethyl sulfide treatment exerted protective effects on CD that were associated with the maintenance of intestinal barrier integrity and the Th/Treg balance. Bis‐2‐(5‐phenylacetamido‐1,2,4‐thiadiazol‐2‐yl) ethyl sulfide treatment may act in part through TCR‐mediated mammalian target of rapamycin complex 1 (mTORC1) signalling activation. In conclusion, inhibition of Gls1 expression attenuated chronic colitis by maintaining intestinal barrier integrity and the Th/Treg balance, thereby ameliorating CD‐like colitis.

## 1 INTRODUCTION

Crohn's disease (CD) is a chronic condition that causes inflammation of the lining of the digestive system with increasing incidence worldwide. All segments of the CD gastrointestinal tract can be affected, causing fibrosis, intestinal obstruction and fistula.^1^ However, the exact aetiology is poorly understood, which limits diagnostic and treatment improvements. The interaction between the intestinal barrier and the immune system may play an important role in the pathogenesis of CD.^2^

Defects in intestinal epithelial barrier function and immune disorders are the characteristic features of CD.^3^, ^4^ Increased permeability of the intestinal barrier can increase the absorption of bacteria, toxins, etc, which generally cannot pass through the normal intestinal mucosa, and stimulate a series of antigen‐specific immune responses and inflammatory changes. Consequently, compensatory immune reactions are excessively triggered a process that is believed to finally result in chronic intestinal inflammation.^5^ Among the variety of inflammatory cells in the intestine, mucosal CD4^+^ lymphocytes are believed to play central roles in both the induction and persistence of chronic inflammation by producing pro‐inflammatory cytokines.^2^, ^6^ Crohn's disease is mainly characterized by an enhanced Th1 response^7^, ^8^ together with an increased Th17 immune response, which plays a critical role in the pathogenesis of CD.^9^ Local immune balance of intestinal tissue is maintained by regulatory T cells (Tregs) in the intestine that inhibit the proliferation and response of other helper‐T cells (Th cells).^10^ Studies have indicated that CD is associated with impaired Treg responses and enhanced Th1/Th17 responses.^11^, ^12^, ^13^ Due to the crucial role of the Th/Treg balance in inducing and sustaining intestinal damage in CD, regulating the balance of immune responses in CD patients may be a new therapeutic strategy.

Previous studies have shown that glutamine (Gln) therapy improves the outcome of experimental colitis by maintaining intestinal barrier integrity and decreasing intestinal permeability through the enhancement of tight junctions in experimental colitis.^14^, ^15^, ^16^ Glutaminase (Gls) is the first enzyme in the glutaminolysis pathway, and it converts Gln to glutamate.^17^ In mammals, there are two different genes encoding Gls, Gls1 (the kidney isoform) and Gls2 (the liver isoform); and Gls1 has greater enzymatic activity than Gls2.^18^ Therefore, we have suggested that inhibition of Gls1 may increase Gln levels and have a protective effect on the intestinal barrier in CD, which has not been reported. Recent research found that targeting Gls1 was efficacious in the treatment of autoimmune diseases. Inhibition of Gls1 by BPTES ([bis‐2‐(5‐phenylacetamido‐1,2,4‐thiadiazol‐2‐yl) ethyl sulfide], a Gls1 specific inhibitor^19^) could decrease Th17 and Th1 responses and increase the Treg response.^19^, ^20^, ^21^, ^22^, ^23^ These observations suggest an essential role of Gls1 in the generation of Th subsets (Th1, Th17 and Treg cells) in autoimmune diseases. We have suggested that Gls1 may play an important role in controlling intestinal T‐cell responses and maintaining intestinal barrier integrity in patients with CD.

Studies on the role of Gls1 in the balance of the Th/Treg‐cell response and experimental colitis in CD have not been reported. Here, we detected increased Gls1 levels in CD patients and *IL‐10*‐deficient (*IL‐10* ^−/−^) mice. The inhibition of Gls1 by BPTES significantly increased Gln expression in *IL‐10* ^−/−^ mice and showed a protective effect in experimental colitis. Furthermore, this study investigated the effects of a Gls1 inhibitor on Th/Treg cell homeostasis, intestinal mucosal inflammation and intestinal barrier integrity in *IL‐10* ^−/−^ mice.

## 2 MATERIALS AND METHODS

### 2.1 Patient specimen preparation

The study was approved by the local ethics committee, and informed consent was obtained from the patients for the use of surgical specimens in this study. Intestinal specimens were collected from patients with CD (n = 13) who underwent intestinal resection, and uninjured bowel was collected from colon cancer patients (control, n = 17). All the CD patients enrolled in this study (seven males, six females; mean age 35.6 [3.2] years; mean BMI 17.9 [0.7] kg/m^2^) had a Montreal classification of A2; L3; B2 and had undergone initial ileocecal resection for stenosis. The patients with colon cancer (11 males, six females; mean age 64.3[5.3] years; mean BMI 18.9 [0.8] kg/m^2^) were enrolled as the control group.

### 2.2 Mice

Both wild‐type (WT) mice and *IL‐10* ^−/−^ mice (C57BL/6J background) were purchased from The Jackson Laboratory and were housed in a specific pathogen‐free (SPF) environment. The *IL‐10* ^−/−^ mice consistently developed colitis at 15 weeks of age when maintained in the SPF environment as previously reported.^24^ The animal experiments were conducted in compliance with the guidelines issued by the China Council for Animal Care and Utilization Committee of Bengbu Medical College (Bengbu, China).

### 2.3 BPTES administration protocol and enteritis symptom assessment

The *IL‐10* ^−/−^ mice (15 weeks old, male) were divided into the control (*IL‐10* ^−/−^, n = 10) and BPTES‐treated groups (*IL‐10* ^−/−^ +BPTES, n = 10); all mice showed spontaneous enteritis. The WT mice (15 weeks old, male, n = 10) were used as negative controls. A total of 60 μg/mice BPTES (Gls1 inhibitor; Selleck Chemicals, USA) or dimethyl sulfoxide (DMSO) in PBS was intraperitoneally administered twice a week for 4 weeks as reported previously.^20^ The *IL‐10* ^−/−^ mice were scored weekly using the inflammatory bowel disease activity index (DAI) and a numerical system, as reported previously.^24^ In short, DAI was calculated by scoring 1 point for the appearance of each of the following features: ruffled fur; occult faecal blood; rectal prolapse <1 mm; and soft stool. An additional point was given for diarrhoea or severe rectal prolapse >1 mm. Therefore, DAI was obtained on a 6‐point (0‐5) scale. Mouse body weight was monitored daily.

### 2.4 Histological examinations

Histologic evaluations were performed on disease activity index‐stained sections of the intestines fixed in 10% formalin solution, as previously described.^25^ In brief, intestinal inflammation was scored on a scale of 0‐4 according to inflammatory cell infiltration in the intestinal lamina propria and changes in the intestinal mucosal architecture. All histological scoring was conducted by two independent pathologists who were blinded to treatment group.

### 2.5 Immunohistochemical analysis

Intestinal Gls1 levels were determined by immunohistochemical analysis as previously described.^26^ Briefly, intestinal tissue sections were deparaffinized, rehydrated, subjected to antigen retrieval, blocked with normal goat serum for 30 minutes and incubated at 4°C overnight with rabbit polyclonal antibody against Gls1 (1:100; Abcam). Then, the samples were incubated for 60 minutes at RT with a 1:300 dilution of biotinylated goat anti‐rabbit immunoglobin G (IgG) antibody (Beyotime, Haimen, China) in PBS, followed by development with DAB (Beyotime). Ten fields from each tissue section were randomly selected to quantify Gls1 expression.

### 2.6 Measurement of Gln

The Gln concentration in the intestinal homogenate was determined using the Gln/glutamate determination kit (GLN‐1; Sigma‐Aldrich, USA) according to the manufacturer's instructions. Absorbance was read at 340 nm using a Tecan Infinite M200 plate reader (Tecan, Austria).

### 2.7 Enzyme‐linked immunosorbent assay

Interleukin‐17A, IFN‐γ and TNF‐α expression levels in the intestine were determined by ELISA. Briefly, intestinal tissue was homogenized in 1 mL of normal saline with protease inhibitors (Sigma‐Aldrich). Then, the homogenates were centrifuged at 1000 *g* at 4°C for 30 minutes, and the supernatant was stored at −80°C until analysis. Interleukin‐17A, IFN‐γ and TNF‐α levels (pg/mg) were measured by ELISA (eBioscience, San Diego, CA).

### 2.8 Immunofluorescence assessment of tight junction proteins

Immunofluorescence analysis of zona occludens‐1 (ZO‐1), occludin and claudin‐1 localization was performed as described previously.^27^ The intestinal frozen sections were cut at 10 μm. After blocking non‐specific background, the sections were incubated with rabbit polyclonal antibody against ZO‐1, occludin and claudin‐1 (Abcam) at 4°C overnight. The corresponding secondary IgG antibodies were fluorescein isothiocyanate (FITC)‐conjugated, and the nuclei were stained with 4,6‐diamidino‐2‐phenylindole (DAPI). Confocal analysis was performed with a confocal scanning microscope (Leica Microsystems; Heidelberg GmbH, Mannheim, Germany).

### 2.9 Intestinal permeability in vivo

After being fasted for 4 hours, the mice were administered FITC‐dextran (4 kDa; Sigma) by gavage at a dose of 600 mg/kg. Then, the mice received isoflurane anaesthesia through inhalation and were killed by spinal dislocation. Blood was collected through cardiac puncture, and serum was isolated using centrifugation. Serum FITC levels were evaluated using fluorometry.^27^

### 2.10 Bacterial translocation

Sterile isolation of mouse liver and spleen was performed for bacteriological cultures. The tissue samples were weighed, and 0.1 g of each sample was homogenized with 0.9 mL of sterile saline. The homogenates were diluted and cultured (100 μL) on MacConkey's agar (Sigma‐Aldrich) at 37°C for 24 hours. Bacterial growth on the plates was expressed as colony forming units/g of tissue, and the presence of more than 10^2^ colonies/g of tissue indicated a positive result.^28^

### 2.11 Flow cytometry

T‐cell responses were analysed by flow cytometry as described previously.^29^ For the Treg analysis, antibodies specific for CD4, CD25 and Foxp3 (eBioscience) were used to analyse the proportion of Tregs in splenocytes and mesenteric lymph node (MLN) cells. For the Th1 and Th17 cell analysis, splenocytes and MLN cells were incubated at 2 × 10^6^ cells/mL in 48‐well plates and stimulated with a cell‐stimulation cocktail (2 μL/well; eBioscience) and Brefeldin A (eBioscience) for 6 hours. The cells were harvested and stained for surface markers with anti‐CD4 and anti‐CD3e antibodies (eBioscience) for 30 minutes at 4°C. After fixation and permeabilization, the cells were incubated with anti‐IFN‐γ or anti‐IL‐17A antibodies (eBioscience) for 1 hour at 4°C. Analyses were performed with a FACSCalibur flow cytometer (BD Biosciences, San Diego, CA), and the data were analysed using FlowJo‐V10 software.

### 2.12 Western blot analysis

Total protein extracts were obtained from intestinal mucosa tissue, and the expression levels of target proteins were analysed by western blot analysis. In short, after SDS‐PAGE, the proteins were transferred to a PVDF membrane, which was immunoblotted with antibodies against Gls‐1, claudin‐1, occludin, ZO‐1, p‐p70 S6K, p70 S6K, p‐4E‐BP1, 4E‐BP1 or β‐actin. Densitometric analysis of protein band intensity was performed with Imagej (National Institutes of Health, USA).

### 2.13 Total RNA extraction and real‐time quantitative PCR

Freshly intestinal mucosa tissues were lysed by Trizol reagent (Invitrogen) and cDNA was generated from 1 μg of isolated RNA using the PrimeScript RT reagent kit with gDNA Eraser (Takara). Real‐time quantitative PCR (qPCR) involved the use of SYBR Green qPCR Mix (Takara). The sequences of specific primers used for qPCR amplification were as follows: mouse Gls‐1 forward/reverse 5′‐GACAACGTCAGATGGTGTCAT‐3′/5′‐TGCTTGTGTCAACAAAACAATGT‐3′. mRNA expression levels were normalized to glyceraldehyde‐3‐phosphate dehydrogenase levels and calculated according to the comparative threshold cycle (Ct) method.

### 2.14 Statistical analysis

Statistical analyses were performed with GraphPad Software (San Diego, CA). Means and SDs were calculated. Binary and categorical data were compared by chi‐squared tests for contingency tables. The parametric Student's *t* test was used to assess the significance of differences between the *IL‐10^−/−^* and *IL‐10^−/−^* +BPTES groups, and differences were considered significant at *P* < 0.05.

## 3 RESULTS

### 3.1 Increased Gls1 expression in the intestines of CD patients and IL‐10^−/−^ mice

**Figure 1:**
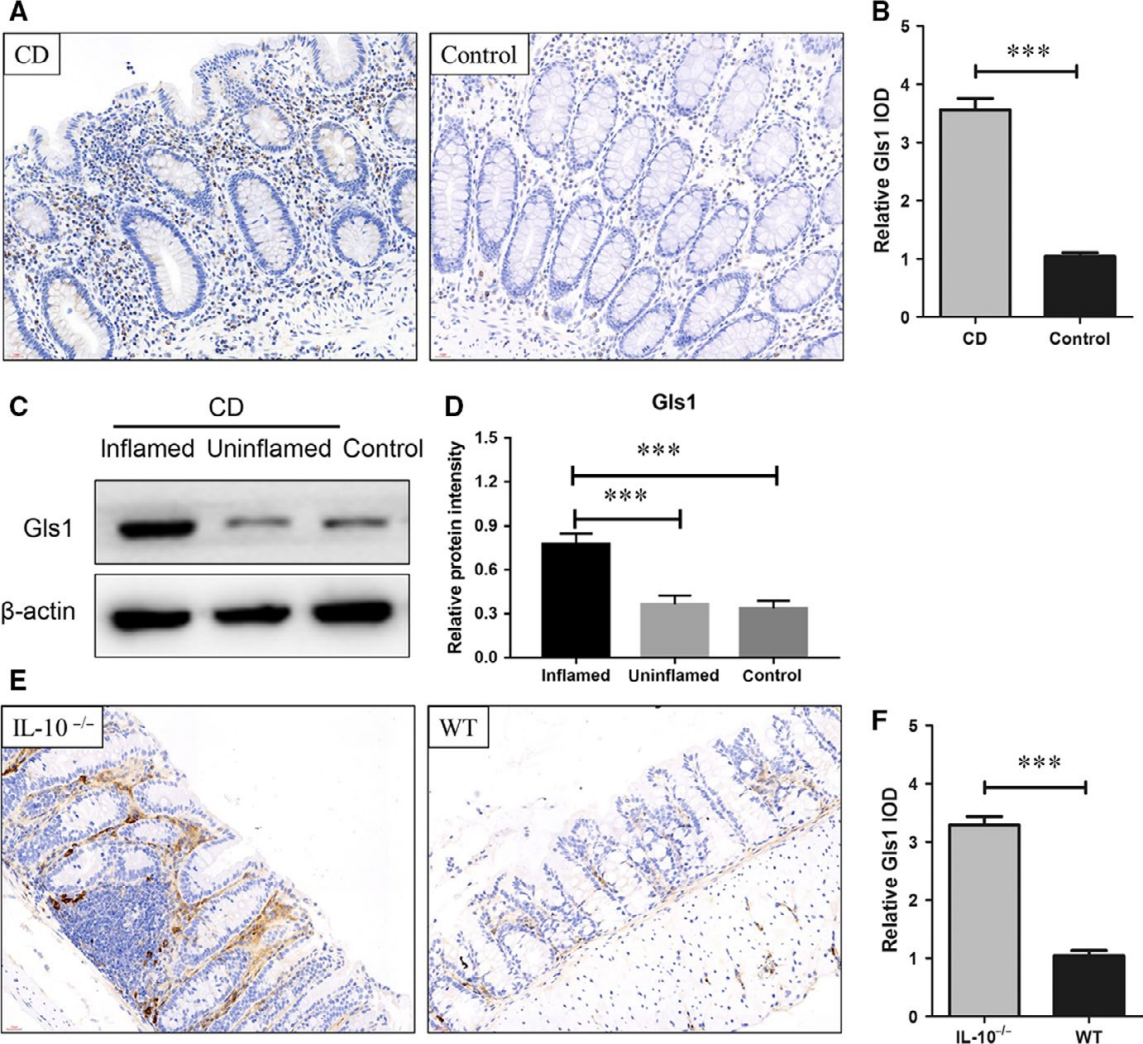
Gls 1 is highly expressed in the intestines of CD patients and *Il‐10* ^–/–^ mice. Immunohistochemical staining with an antibody that recognizes Gls1 was performed on the intestines of control and CD patients (A). The quantitative analysis presented in (B) shows Gls1 expression in the intestines of CD (n = 13) and control patients (n = 17). (C,D) Western blot analysis of Gls1 in the intestinal mucosa in the intestines of CD patients (inflamed and uninflamed areas) and control patients. (E,F) The expression of Gls1 in the intestines of *Il‐10^–/–^* mice and WT mice (n = 8 in each group). CD, Crohn's disease; Gls1, glutaminase 1; IOD, integrated optical density; WT, wild‐type. The data are presented as the relative IOD ± SD. ***P < 0.001

The presence of Gls1 can aggravate inflammatory reactions in autoimmune diseases. We found significantly increased Gls1 expression in intestinal mucosa tissue of CD patients compared with control patients (Figure 1A,B). We also analysed the correlation of CD patients' clinical parameters with Gls1 expression, but we did not find a definite correlation. Glutaminase 1 expression was also higher in the inflamed areas of CD patients' intestinal tissue than in the uninflamed areas (Figure 1C,D). In the animal experiments shown in Figure 1C,D, the data indicated higher Gls1 expression in the intestinal mucosa of *IL‐10^−/−^* mice than in that of WT mice. The increased Gls1 expression in the intestinal tissues of CD patients and *IL‐10^−/−^* mice suggest that Gls1 may be related to the development of CD.

### 3.2 Systemic delivery of the Gls1 inhibitor (BPTES) ameliorates experimental colitis in IL‐10^−/−^ mice

**Figure 2:**
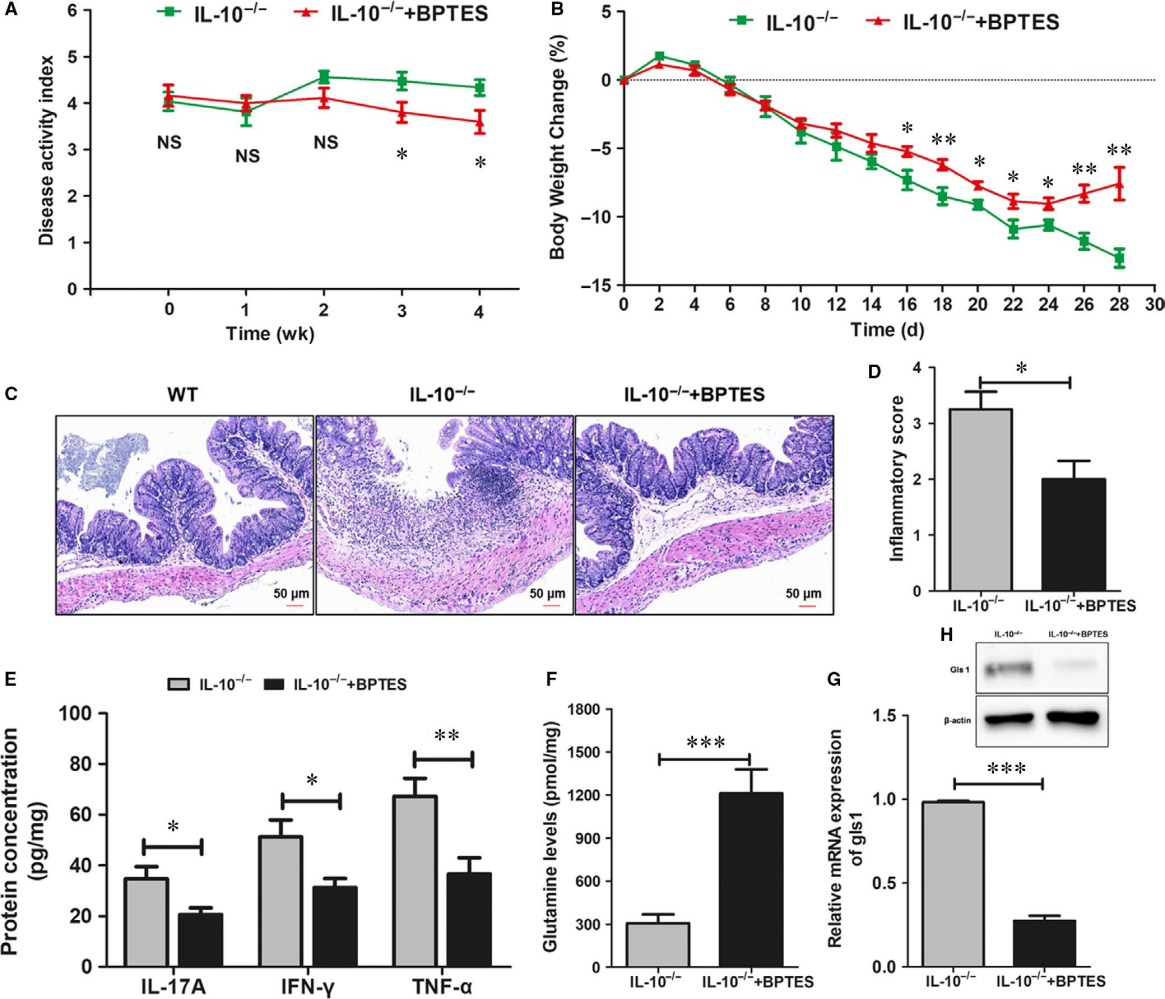
Systemic delivery of BPTES ameliorates experimental colitis in *Il‐10* ^–/–^ mice. The 15‐wk‐old *Il‐10* ^–/–^ mice were intraperitoneally treated with BPTES (60 μg/mice) or DMSO in PBS for 4 wk. A, The disease activity index (DAI) in BPTES‐treated *Il‐10* ^–/–^ mice and untreated *Il‐10* ^–/–^ mice was scored weekly. B, Bodyweight changes in the BPTES‐treated *Il‐10* ^–/–^ mice and untreated *Il‐10* ^–/–^ mice. C, Intestinal tissue sections were stained with haematoxylin and eosin for histological analysis under light microscopy (scale bar: 50 μm). The histological inflammation score (D) was analysed as described in the Section 22. E, ELISAs of IL‐17A, TNF‐α and IFN‐γ protein expression in intestinal tissue homogenate. F, Concentration of Gln in intestinal homogenate. (G,H) The relative mRNA and protein levels of Gls1 in intestinal mucosa. The data are expressed as the mean ± SD (n = 8 for each group). *P < 0.05, **P < 0.01, ***P < 0.001. BPTES, bis‐2‐ (5‐phenylacetamido‐1,2, 4‐thiadiazol‐2‐yl) ethyl sulphide; Gls1, glutaminase 1; IFN, interferon; IL, interleukin; TNF, tumour necrosis factor

We used BPTES to inhibit Gls1 expression and explored the role of Gls1 in enteritis in *IL‐10^−/−^* mice in the following study. BPTES or DMSO was administered intraperitoneally twice a week in *IL‐10^−/−^* mice. Our data showed that BPTES‐treated *IL‐10^−/−^* mice had a lower mean DAI than DMSO‐treated *IL‐10^−/−^* mice beginning at the third week after drug administration (Figure 2A). Body weights decreased in both groups throughout the study; however, compared with DMSO‐treated *IL‐10^−/−^* mice, BPTES‐treated mice showed a significantly less body weight loss (Figure 2B) from day 16 to the end of the experiment. Meanwhile, the histological inflammation score for the intestinal tissues was significantly decreased in BPTES‐treated mice compared with DMSO‐treated *IL‐10^−/−^* mice (Figure 2C,D). Moreover, the levels of inflammatory factors (IL‐17A, TNF‐α and IFN‐γ) were decreased in the intestinal tissues of BPTES‐treated *IL‐10^−/−^* mice compared with control *IL‐10^−/−^* mice (Figure 2E). Our data also demonstrated that BPTES could significantly inhibit Gls1 expression and increase Gln levels in intestinal tissues (Figure 2F‐H), consistent with previous studies.^20^, ^23^ Consequently, BPTES treatment was found to ameliorate the signs of experimental colitis in *IL‐10^−/−^* mice.

### 3.3 Treatment with BPTES improves intestinal barrier function in IL‐10^−/−^ mice

**Figure 3:**
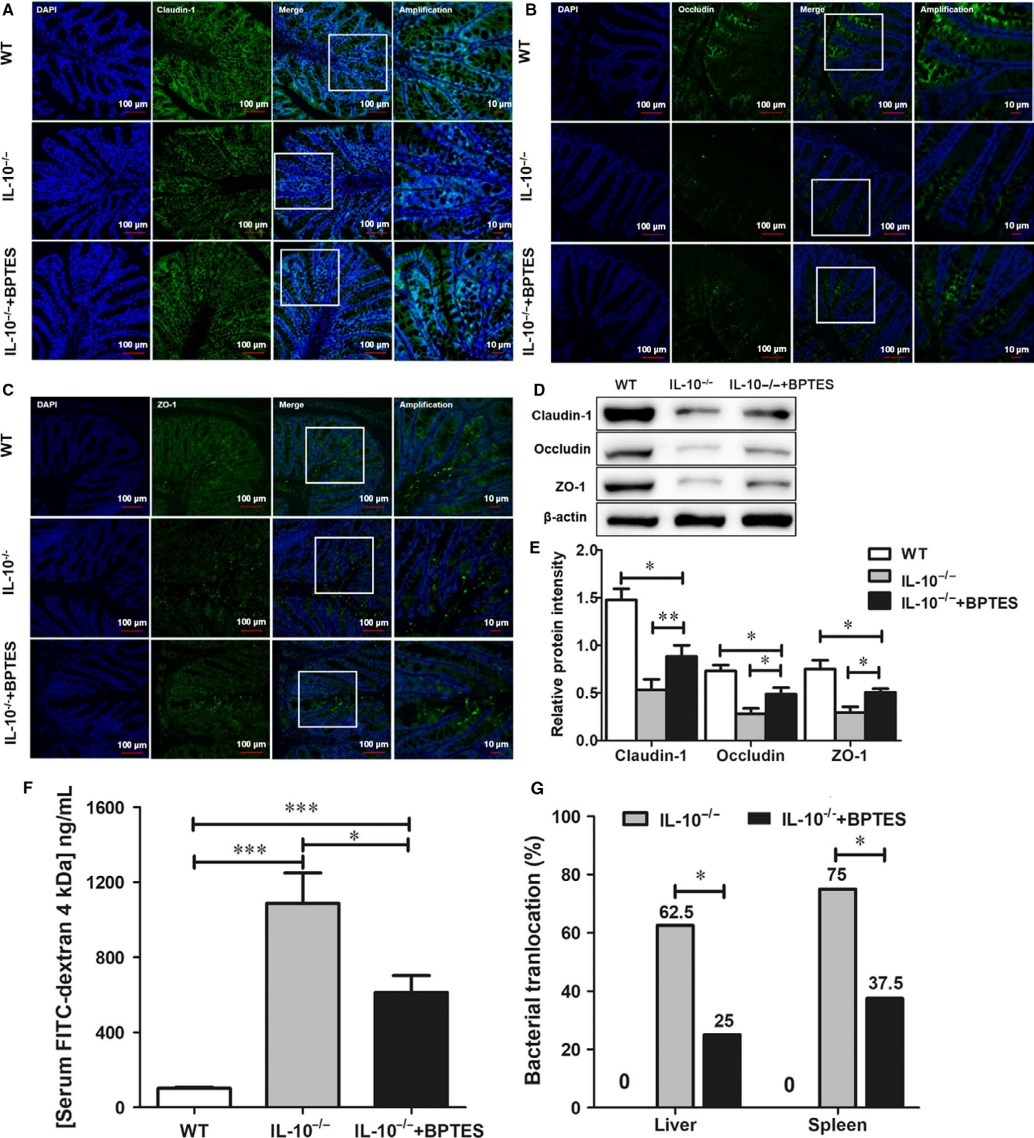
BPTES treatment improves intestinal barrier function in *IL‐10^−/−^* mice. The localization of claudin‐1, occludin, ZO‐1 and DAPI (DNA) in intestinal sections from the three groups was analysed by immunofluorescence. Tight junction protein (green; ZO‐1, occludin and claudin‐1) and DAPI (blue) signals were merged, as were amplified tight junction protein and DAPI images (A‐C). Western blot analysis of claudin‐1, occludin and ZO‐1 levels in the intestinal mucosa of WT, *IL‐10^−/−^* and BPTES‐treated *IL‐10^−/−^* mice (D,E). Evaluation of intestinal barrier permeability by administering FITC‐dextran (4 kDa; Sigma) at a dose of 600 mg/kg. F, Serum FITC levels were evaluated using fluorometry. G, The rate of bacterial translocation in the liver and spleen was determined by bacteriological cultures as described in the Section 22. The data are expressed as the mean ± SD (n = 8 per group). BPTES, bis‐2‐ (5‐phenylacetamido‐1,2, 4‐thiadiazol‐2‐yl) ethyl sulphide; DAPI, 4,6‐diamidino‐2‐phenylindole; FITC, fluorescein isothiocyanate; NS, no significance; ZO‐1, zona occludens‐1. *P < 0.05, **P < 0.01, ***P < 0.001

Intestinal barrier dysfunction is one of the suggested causes of CD pathogenesis, and tight junction proteins (including claudin‐1, occludin and ZO‐1) contributing to barrier function; decreased levels of those proteins lead to altered tight junction structure. Thus, immunofluorescence was used to evaluate the intestinal mucosa of BPTES‐treated mice and control mice. The results are shown in Figure 3A‐C. The expression levels of claudin‐1, occludin and ZO‐1 were significantly decreased in *IL‐10^−/−^* mice compared with WT mice. However, in *IL‐10^−/−^* mice, tight junction protein expression was obviously increased after BPTES treatment compared with control, although these protein levels were still lower than those in WT mice. Western blot data confirmed the above results (Figure 3D‐E). Damaged structures can lead to intestinal barrier dysfunction, which can alter intestinal permeability. Bis‐2‐(5‐phenylacetamido‐1,2,4‐thiadiazol‐2‐yl) ethyl sulfide‐treated mice showed lower levels of serum dextran conjugates than control mice (*P* < 0.01; Figure 3F). Bacterial translocation plays an important role in the aetiology of human inflammatory bowel disease.^30^ Bacterial culture was used to detect bacterial ectopic conditions in the mouse liver and spleen (Figure 3G). The rates of bacterial translocation in the liver (*P* < 0.001) and spleen (*P* < 0.001) were lower in BPTES‐treated mice than in DMSO control mice. These findings demonstrated that intestinal barrier dysfunction can be alleviated by BPTES treatment.

### 3.4 BPTES treatment maintains the Th/Treg balance in IL‐10^−/−^ mice

**Figure 4:**
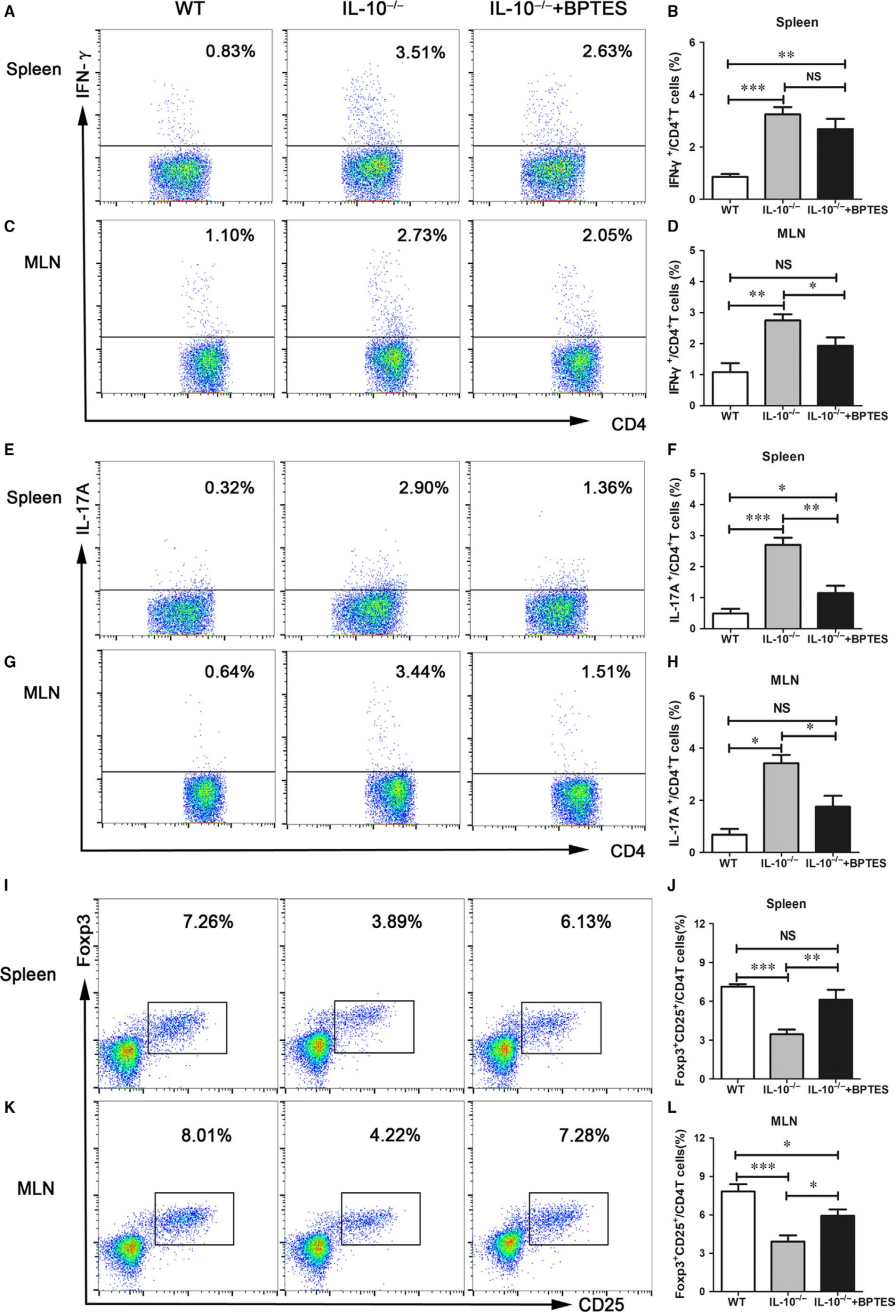
BPTES treatment maintains the Th/Treg balance in *IL‐10^−/−^* mice. Splenocytes and mesenteric lymph node cells from each group were prepared at the fourth week after drug administration. IFN‐γ‐producing CD4^+^ T cells and IL‐17A‐producing CD4^+^ T cells were quantified by intracellular cytokine staining as described in the Section 22. (A,C) Representative flow cytometric images of IFN‐γ‐producing CD4^+^ T cells. Summary of the percentage (B,D) of IFN‐γ‐producing CD4^+^ T cells. (E,G) Representative flow cytometric images of IL‐17A‐producing CD4^+^ T cells. Summary of the percentage (F,H) of IL‐17A‐producing CD4^+^ T cells. (I,J) Representative flow cytometric images of CD4^+^ CD25^+^ Foxp3^+^ T cells in different organs. Summary of the percentage (K,L) of CD4^+^ CD25^+^ Foxp3^+^ T cells. The data are expressed as the mean ± SD (n = 8 per group). BPTES, bis‐2‐ (5‐phenylacetamido‐1,2, 4‐thiadiazol‐2‐yl) ethyl sulphide; IFN, interferon; IL, interleukin; NS, no significance; *P < 0.05, **P < 0.01, ***P < 0.001

The excessive activation of Th1 and Th17 cells plays a critical role in the pathogenesis of CD. Furthermore, previous studies indicated that Gls1 could regulate Th1‐ and Th17‐cell responses in autoimmune diseases.^30^ Therefore, we performed intracellular cytokine staining and flow cytometric analysis to confirm the effect of Gls1 on Th1 and Th17 cells in *IL‐10^−/−^* mice. The data in Figure 4 show that the proportion of IFN‐γ^+^ CD4^+^ T cells (Th1 cells) in the spleen and MLNs was significantly decreased in BPTES‐treated mice compared with control mice (Figure 4A‐D). Meanwhile, flow cytometric data showed a significant decrease in the proportion of IL‐17A^+^ CD4^+^ T cells (Th17 cells) in BPTES‐treated mice compared with control mice (Figure 4E‐H). The results confirmed that Gls1 promotes Th1‐ and Th17‐cell responses, and BPTES treatment can inhibit Th1 and Th17 responses in experimental colitis in *IL‐10^−/−^* mice. We know that CD4^+^ CD25^+^ Foxp3^+^ T cells (Tregs) have extensive immunosuppressive effects on other Th cells^10^, ^31^; therefore, we further examined Tregs in the spleen and MLNs of BPTES‐treated and control mice (Figure 4I‐L). Despite the impaired response of Tregs in BPTES‐treated mice compared with WT mice, the proportion of Tregs in BPTES‐treated mice was significantly higher than that in *IL‐10^−/−^* mice in both the spleen and MLNs. These data indicated that BPTES was associated with the maintenance of the Th/Treg balance, which contribute to controlling experimental colitis in *IL‐10^−/−^* mice.

### 3.5 Gls1 regulates different T‐cell subset responses in *IL‐10^−/−^* mice involved in activating mTORC1

**Figure 5:**
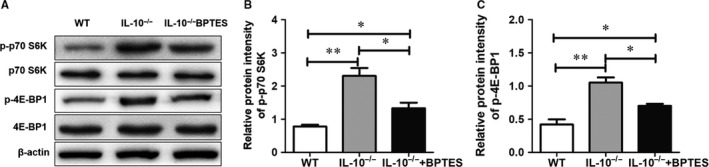
Gls1 regulate different T‐cell subset responses in *IL‐10^−/−^* mice involve inactivating mTORC1. Total protein extracts were prepared from intestinal tissue, and the protein levels of p‐p70 S6K, p70 S6K, p‐4E‐BP1 and 4E‐BP1 were analysed by western blot; β‐actin was used as an internal reference A,. Densitometric assays of all protein band intensities (B,C). The data are expressed as the mean ± SD (n = 8 per group). Gls1, glutaminase 1; NS, no significance; *P < 0.05, **P < 0.01

In T cells, it has been reported that Gln transporter‐deficient T cells have a decreased Th1/Th17 response and reduced TCR‐mediated mammalian target of rapamycin complex 1 (mTORC1) activity.^32^ In addition, inhibition of Gls1 can reduce the phosphorylation of p70 S6K, which is downstream of mTORC1.^20^ We have suggested that Gls1 up‐regulates Th1/Th17 and down‐regulates the Treg response involved in regulating mTORC1 activity. As expected, BPTES treatment down‐regulated p70 S6K (Figure 5A,B) and 4E‐BP1 (Figure 5C) phosphorylation in the intestines of *IL‐10^−/−^* mice.

## 4 DISCUSSION

In this study, we detected significantly increased Gls1 expression on the CD intestinal mucosa and identified the protective effect of Gls1 in a spontaneous mouse model of chronic colitis. We demonstrated that Gls1 inhibition by BPTES ameliorated colitis in *IL‐10^−/−^* mice, as shown by histopathology and by reductions in inflammation scores and the DAI, which was associated with significantly decreased Th1 and Th17 responses but increased Treg responses. Furthermore, we found that the regulatory effects of Gls1 on the T‐cell subset response may involve mTORC1 activation.

Previous studies have shown that the glutaminolysis pathway plays an important protective role in experimental colitis models and that targeting Gls1 (the first enzyme in the glutaminolysis pathway) is an effective therapy for autoimmune diseases.^16^, ^20^, ^22^, ^23^, ^33^ In this study, Gls1 was highly expressed in the intestines of CD patients and *IL‐10^−/−^* mice. We suspect that high Gls1 levels in the intestine of CD patients may be related to the disease extent. Indeed, we found that inhibiting Gls1 expression in *IL‐10^−/−^* mice could ameliorate experimental colitis, which was associated with reduced mean DAI, body weight loss, histological inflammation scores and pro‐inflammatory mediator expression. An excessive inflammatory response could contribute to intestinal barrier defects, and CD patients often suffer from impairments in the intestinal mucosal barrier.^1^, ^3^ Previous research has reported that Gln supplementation can improve intestinal barrier integrity and intestinal permeability by enhancing tight junctions in experimental colitis.^15^, ^34^ In our study, we found that the inhibition of Gls1 by BPTES could ameliorate the damage in the intestinal barrier structure in *IL‐10^−/−^* mice, increase the expression of tight junction proteins (including claudin‐1, occludin and ZO‐1) and decrease intestinal permeability.

Because of the different roles of Th1/Th17 cells and Tregs in CD, the balance between pro‐inflammatory cells (Th1 and Th17) and anti‐inflammatory cells (Treg) can control immune‐mediated pathology and consequent tissue damage in CD.^35^, ^36^ Our results and previous research showed an imbalance between Tregs and Th1 and Th17 cells in *IL‐10^−/−^* mice, in which increased Th1 and Th17 responses and decreased Treg responses were observed compared to WT mice. In the present study, we focused on the immunoregulatory role of metabolism on T‐cell differentiation. Targeting Gls1 had a therapeutic effect on experimental autoimmune encephalomyelitis (EAE) and rheumatoid arthritis, mainly by decreasing Th17 and Th1 responses and increasing the Treg response.^19^, ^20^, ^21^, ^22^, ^23^ We found that the inhibition of Gls1 by BPTES induced a significantly increased Treg response and decreased Th1 and Th17 responses in both a circulatory immune organ (spleen) and a local immune organ (MLN) compared with the control. Although the mechanism by which Gls1 influences T‐cell subset responses remains unclear, several studies have shed light on this issue. Glutamine has been reported to enhance mTORC1 signalling, which supports the differentiation of naïve cells into Th1 cells and inhibits Treg‐cell differentiation.^37^ In addition, Michihito Kono et al reported that the inhibition of Gls1 reduced p70 S6K phosphorylation, Th17 differentiation and the severity of EAE.^20^ Consistent with these studies, our findings confirmed that Gls1 regulates different T‐cell subset responses by activating mTORC1 signalling in a mouse CD model.

Our findings have certain clinical implications. Glutamine plays a protective role in the intestinal barrier.^14^ Our study found that Gls1 enhances Gln levels to protect against colitis and directly regulates the immune response, promotes the Th1/Th17 response and inhibits the Treg response, thus reducing intestinal damage. In addition, CD patients generally exhibit diarrhoea, and oral Gln may not be effectively absorbed,^4^ whereas Gls1 plays an important role in protecting against enteritis by inhibiting Gln degradation.

Our study has some limitations. First, our data indicated that Gls1 expression was significantly higher in the intestine of CD patients than in normal tissue, but we did not investigate the possible mechanisms leading to this elevation. Second, the inhibition of Gls1 showed that the protective effect against colitis may occur through other pathways in addition to regulating the balance of Th/Treg responses. Finally, Gls1 regulates T‐cell responses possibly through mechanisms other than mTORC1 activation.

In summary, our study demonstrated that Gls1 expression is increased in CD patients' intestinal mucosa. Inhibition of Gls1 by BPTES maintains the Th/Treg balance, controls inflammatory reactions and maintains intestinal barrier integrity, which ameliorates spontaneous colitis in *IL‐10^−/−^* mice. We suggest that the therapeutic effect of Gls1 on CD‐like colitis is mediated at least partially through regulation of the T‐cell subset response involved in the mTORC1 signalling pathway.

## CONFLICT OF INTEREST

The authors disclose no potential conflict of interest.

## AUTHOR CONTRIBUTION

J. Li and L. Zuo contributed to the study design, experiments, data analysis and drafting of the manuscript. J. Hu designed the experiments. Y. Tian, Y. He, Z. Zhang, P. Guo and Y. Ge contributed technical support and scientific advice and assisted with the manuscript revision. All the authors read and approved the final manuscript.

## DATA AVAILABILITY STATEMENT

The data used to support the findings of this study are included in the article.
